# Biofeedback pelvic floor muscle training versus posterior tibial nerve electrostimulation in treatment of functional obstructed defecation: a prospective randomized clinical trial

**DOI:** 10.1186/s43166-022-00148-8

**Published:** 2022-09-23

**Authors:** Emmanuel Kamal Aziz Saba, Mervat Sheta Elsawy

**Affiliations:** Physical Medicine, Rheumatology and Rehabilitation Department, Faculty of Medicine, Alexandria University, Alexandria, Alexandria Governorate Egypt

**Keywords:** Biofeedback, Biofeedback pelvic floor muscle training, Posterior tibial nerve electrostimulation, Functional obstructed defecation, Obstructed defecation

## Abstract

**Background:**

Functional obstructed defecation is a common anorectal problem among adult population. The objective was to compare the short-term efficacy of biofeedback pelvic floor muscle training versus transcutaneous posterior tibial nerve electrostimulation in treatment of patients with functional obstructed defecation.

**Results:**

There were 41 patients completed the study. There were no statistical significant differences between biofeedback pelvic floor muscle training group and transcutaneous posterior tibial nerve electrostimulation group regarding different clinical characteristics, as well as, electrophysiological findings. There was statistically significant reduction in all outcome measures after intervention in both groups. The primary outcome measure was Modified obstructed defecation score. Secondary outcome measures were Patient Assessment of Constipation-Quality of Life questionnaire, time of toileting, and maximum anal pressure during straining to evacuate. No significant differences were present between both groups regarding different outcome measures in the pretreatment and post-treatment assessments. Successful outcome was reported in 81% of patients in biofeedback pelvic floor muscle training group in comparison to 40% of patients in the posterior tibial nerve electrostimulation group according to the Modified obstructed defecation score which was the primary outcome measure.

**Conclusions:**

Both biofeedback pelvic floor muscle training and posterior tibial nerve electrostimulation are considered effective methods in the treatment of functional obstructed defecation. However, biofeedback pelvic floor muscle training seems to be more effective and superior in comparison to posterior tibial nerve electrostimulation. Posterior tibial nerve electrostimulation could be combined with biofeedback pelvic floor muscle training or considered as a second line therapy after failure of biofeedback pelvic floor muscle training.

**Trial registration:**

Pan African Clinical Trials Registry, PACTR202009762113535. Registered 2 September 2020—retrospectively registered, https://pactr.samrc.ac.za/TrialDisplay.aspx?TrialID=12321.

## Background

Obstructed defecation (OD) is a common anorectal problem as it occurs in about 7% of the adult population [1, 2]. It is characterized by difficulty or inability to defecate following the urge for defecation, feeling of incomplete evacuation with excessive straining and/or performing manual maneuvers to promote evacuation in more than 25% of defecation attempts [3, 4]. There are two forms of OD which represent different pathophysiological mechanisms. They are either functional OD as anismus or mechanical OD due to structural lesions as rectocele and rectal intussusception [5, 6].

Treatment of OD includes a diversity of tools [7]. All of them aim to improve the symptoms of the patient and improve patient’s quality of life [8, 9]. These include conservative treatment and surgical treatment. Treatment starts by using conservative measures which include dietary modification, life style modification, and laxatives in addition to biofeedback pelvic floor muscle training (BF) and posterior tibial nerve electrostimulation (PTNS) [9, 10]. In case of mechanical OD due to anatomical lesions, surgical restoration of normal anatomy can be used [8, 9].

Biofeedback therapy appears to have a long lasting effect. It is very effective for patients suffer of functional OD [11]. It is the initial therapy for functional OD after failure of dietary modification, life style modification, and laxatives [12, 13]. If BF training failed to improve the condition, PTNS was applied as a sort of peripheral neuromodulation [14, 15]. The objective of the research was to compare the short-term efficacy of BF training versus transcutaneous PTNS in treatment of patients with functional OD.

## Methods

**Fig. 1:**
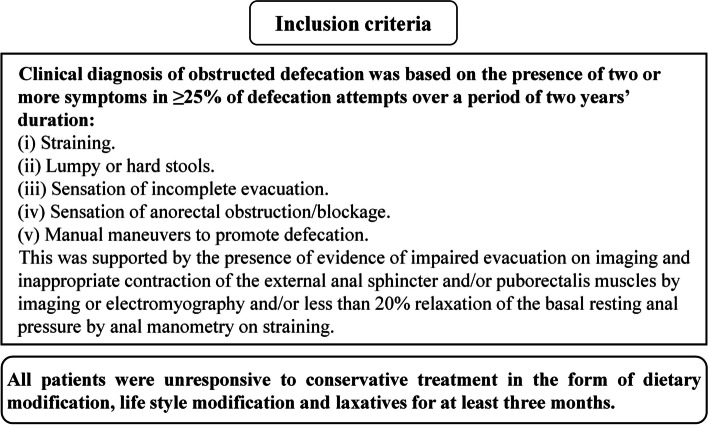
Study inclusion criteria [4, 5]

**Fig. 2:**
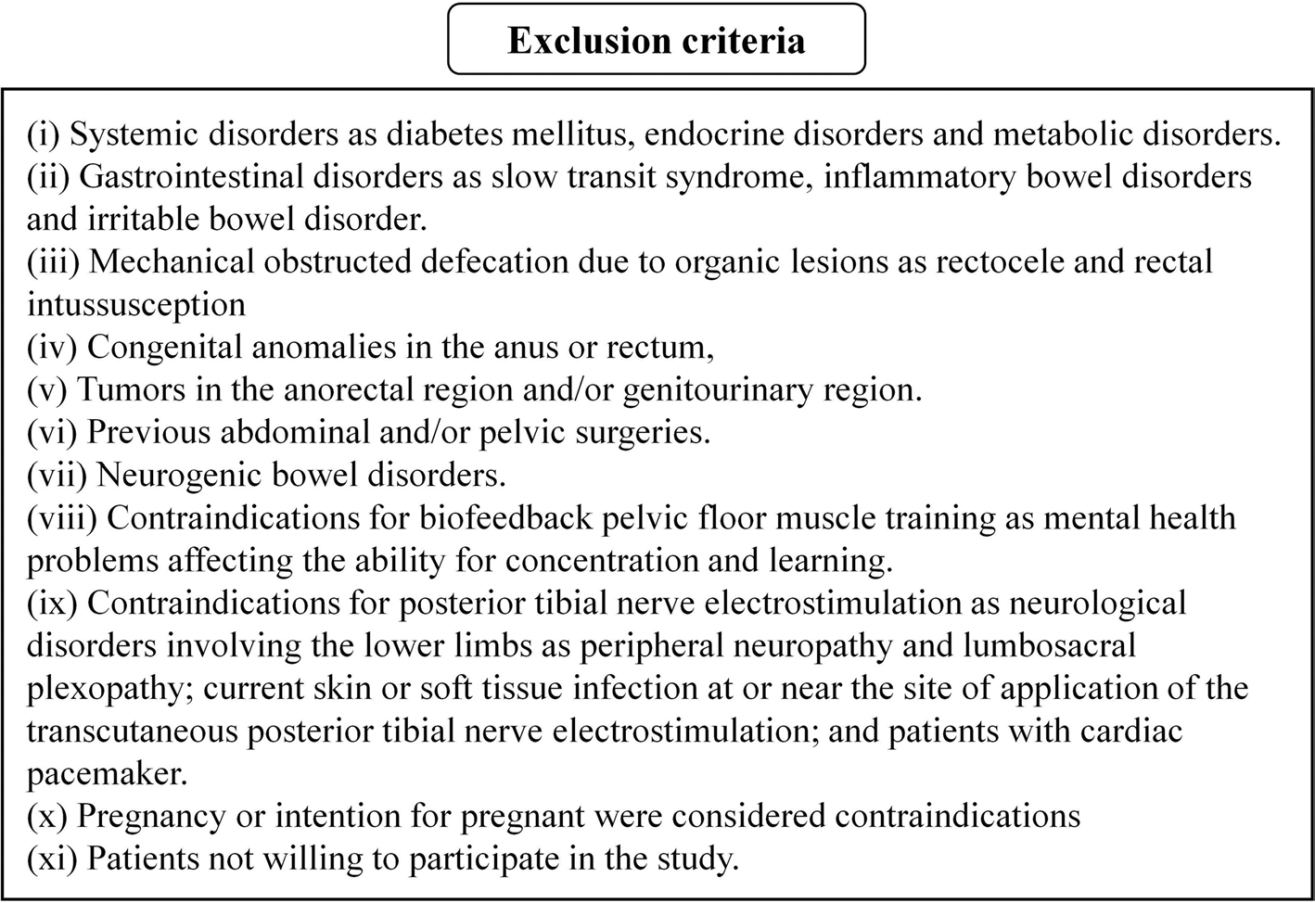
Study exclusion criteria [15, 16]

This prospective study included randomly selected patients with functional OD from those attending the Pelvic Floor Rehabilitation clinic between August 2018 and September 2020. The inclusion criteria and exclusion criteria of the study are illustrated in Figs. 1 and 2 [4, 5, 15, 16]. All the included patients were unresponsive to dietary modification, life style modification, and laxatives for a period of at least 3 months. Patient withdrawal or lost to follow-up were excluded from the analysis. Explanation of the study to the patients was associated with giving an informed consent by each patient. Ethics Committee of the faculty sanctioned the research. The study adhered to CONSORT guidelines. The research was registered in Pan African Clinical Trials Registry (a trial registry) with an identifier number of PACTR202009762113535.

**Fig. 3:**
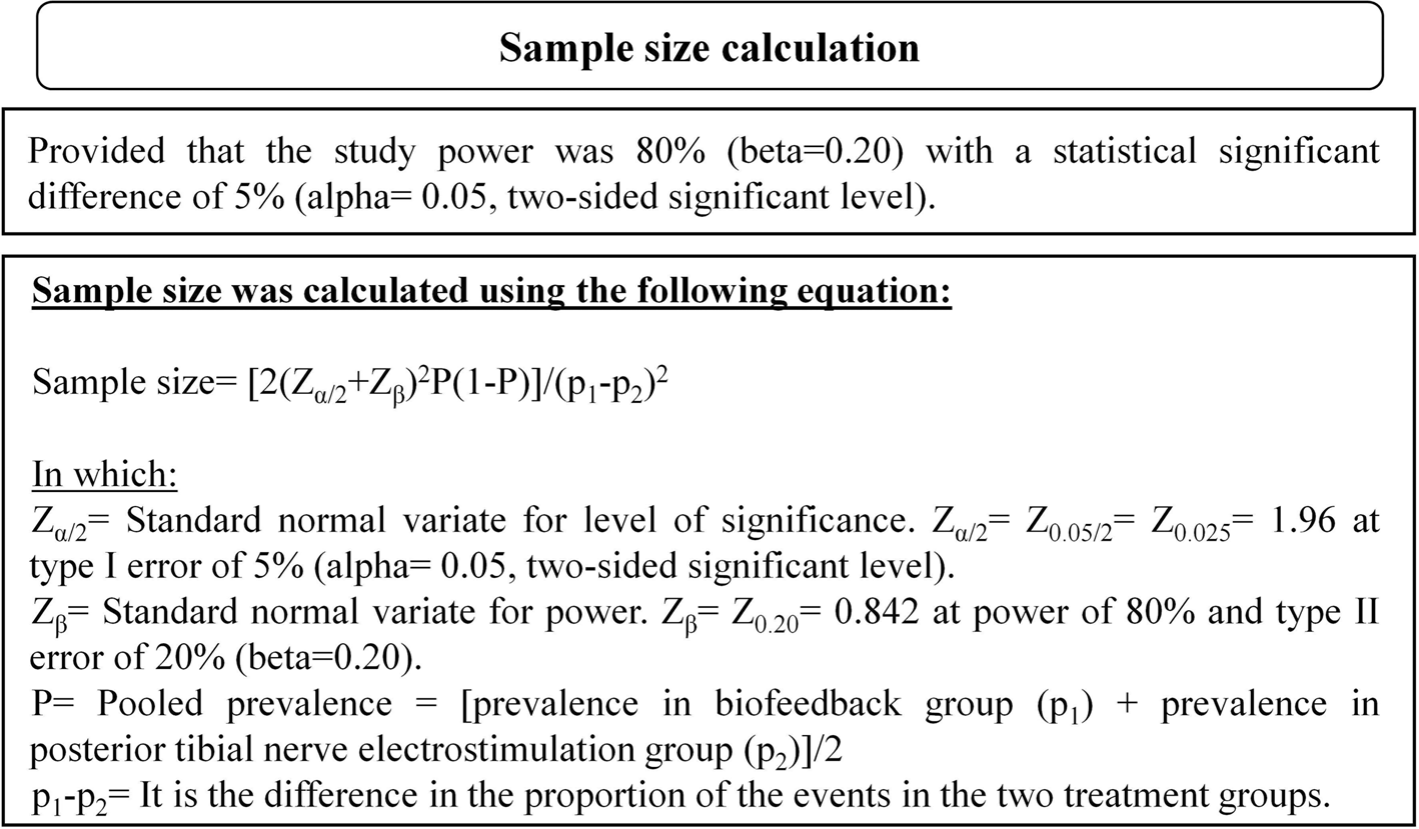
Sample size calculation [19, 20]

Sample size was calculated depending on data of previous studies [17, 18]. The proportion of patients improved with BF and the proportion of patients improved with PTNS were used [17, 18]. The study power was 80% (beta = 0.20) with a statistical significant difference of 5% (alpha = 0.05, two-sided significant level). Depending on these data, the size of the sample was calculated using the equation for sample size calculation illustrated in Fig. 3 [19, 20]. The estimated sample size was at least 16 patients per treatment group. There was an estimation of about 10% of the sample size might be lost for follow-up [21]. So, at least 18 patients per treatment group had been recruited to ensure proper sample size to achieve significant level.

The study included 59 randomly selected patients with functional OD. Exclusion criteria were present in 17 patients who had been excluded. Forty-two patients participated in the trial. One patient had been lost to follow up. This was secondary to the COVID-19 pandemic lock down [22]. This patient was excluded from the analysis.

**Fig. 4:**
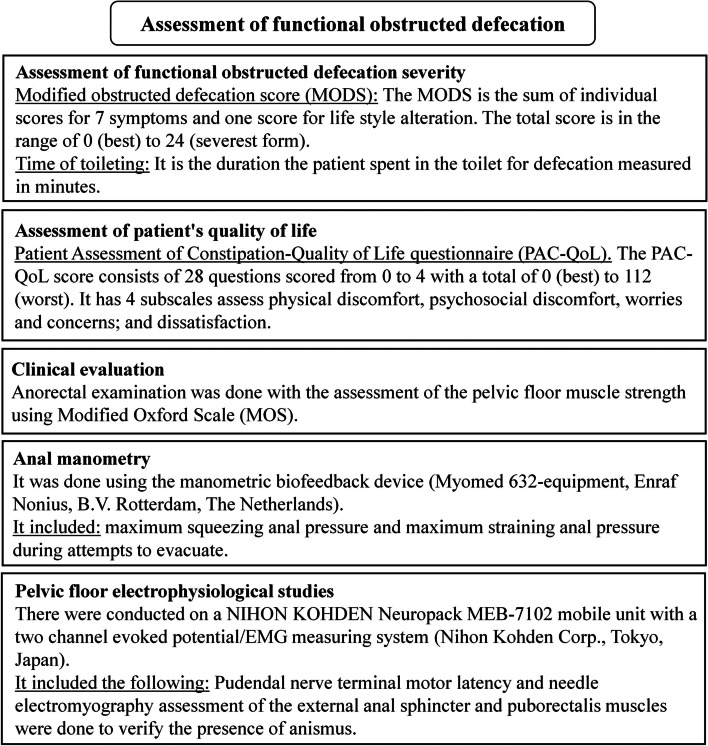
Illustration shows different aspects of assessment of functional obstructed defecation [1, 13, 15, 24–27]

Patients involved in the study were assessed as the following: demographic data collection, history taking and body mass index calculation [23]. Figure 4 illustrates different aspects of assessment of functional OD. Assessment of functional OD severity was done by using Modified OD score (MODS) and time of toileting (Fig. 4) [15, 24]. The patient's quality of life assessment was done by using the Patient Assessment of Constipation-Quality of Life questionnaire (PAC-QoL) (Fig. 4) [24]. Clinical evaluation was performed to all patients (Fig. 4) [25]. Anal manometry assessment was done (Fig. 4) [1, 13]. Pelvic floor electrophysiological studies were conducted to assess the pudendal nerve terminal motor latency and electromyography for the external anal sphincter (EAS) and puborectalis (PR) muscles to verify the presence of anismus as a diagnosis of functional OD [26, 27]. Electromyographic features of anismus was the presence of inappropriate paradoxical contraction or failure of complete relaxation of the EAS and/or PR muscles during simulated defecation (straining to defecate) [26, 27].

Patients were instructed to stop medications during the clinical trial. Also, they were instructed to maintain normal diet. They were ordered to fill a bowel diary to report the occasional use of laxatives or defecation assistance maneuvers like using of suppositories, enemas, and/or digitation of the rectum [15].

The patients were randomly distributed to receive either BF (BF group) or PTNS (PTNS group). They were distributed by one of the researcher. The allocation was performed on an equal basis of 1:1 ratio with randomly permuted block sizes of variable length (two and four). (A) BF group: it constituted of 21 patients. The patients received 12 sessions of BF training at a frequency of two sessions per week over a period of 6 weeks. (B) PTNS group: it constituted of 21 patients. The patients received 18 sessions of transcutaneous PTNS at a frequency of three sessions per week over a period of 6 weeks.

At the initial session, all patients received health education. It consisted of illustration of pelvic floor anatomy with explanation of defecation physiology. It included advice about high fiber diet and fluid intake with regular bowel habits and defecation behavior [28]. Also, patients were instructed to practice pelvic floor exercises (strengthening Kegel exercises) and to practice relaxation during defecation attempts [16].

**Fig. 5:**
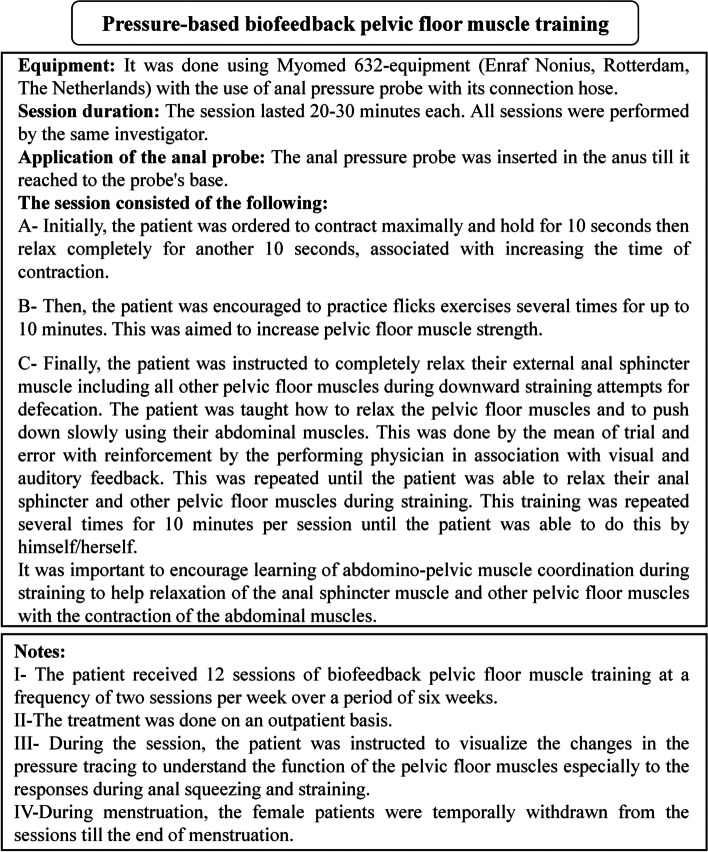
Technique of pressure-based biofeedback pelvic floor muscle training [13, 16, 29]

**Fig. 6:**
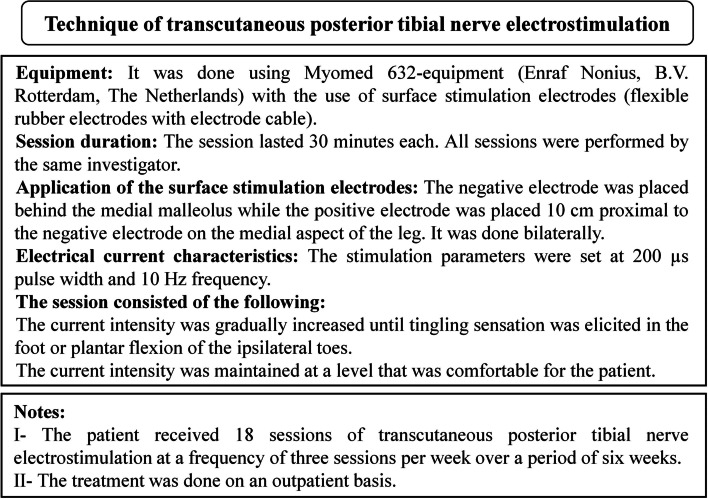
Technique of transcutaneous posterior tibial nerve electrostimulation [15, 30]

The technique of pressure-based BF training is demonstrated in Fig. 5 [13, 16, 29]. The technique of transcutaneous PTNS is demonstrated in Fig. 6 [15, 30].

The initial baseline assessment was done before starting the treatment. Reassessment of the patients after intervention was done at the end of the 6-week session. It included assessment questionnaires (MODS and PAC-QoL questionnaire), time of toileting, and anal manometry assessment of maximum anal pressure during straining to evacuate and maximum squeezing anal pressure [1, 13].

The outcome measures were the following: (i) primary outcome measure was MODS [23]. (ii) Secondary outcome measures were PAC-QoL, time of toileting and maximum anal pressure during straining to evacuate [13, 24]. According to the results of the outcome measures in the post-treatment evaluation, the participants were grouped as having (i) improvement: if the patient had at least 50% improvement in the outcome measure after therapy. (ii) No improvement: if the patient had less than 50% improvement in the outcome measure after therapy [30, 31].

**Fig. 7:**
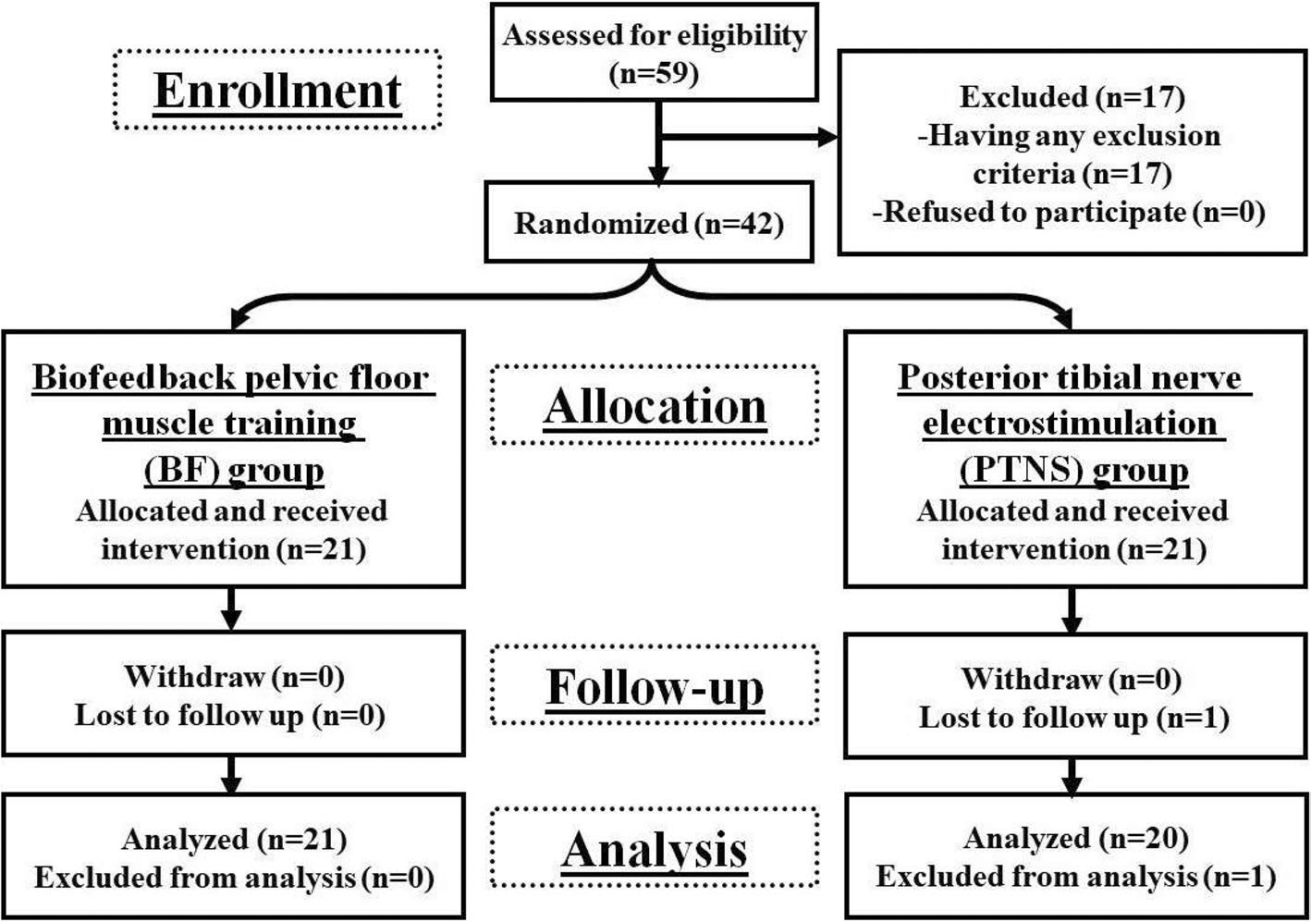
Study profile. n, number of patients

The current study was not blinded. Figure 7 is an illustration of the study profile. Aiming to avoid bias in the measurement of outcome measures, the pretreatment assessment and the intervention were done by one of the authors and the post-treatment assessment was done by the other author.

Statistical Package of Social Science (version 17) software was used. Mann-Whitney test, Wilcoxon signed ranks test, chi-square test, and Fisher’s exact test (when required) were performed. Significance was reported to any *P* ≤ 0.05.

## Results

The study includes 42 patients. However, there was one patient from the PTNS group was lost to follow up. This patient was excluded from the analysis. Subsequently, there were 41 patients (25 females [58.53%]) completed the study. Their mean age was 37.95 ± 15.45 years (ranged from 19 to 80 years). They were randomly distributed to receive either BF (BF group) or PTNS (PTNS group). The BF group consisted of 21 patients (51.2%) while PTNS group consisted of 20 patients (48.8%). No significant differences were found between both groups regarding baseline characteristics, as well as, the electrophysiological findings (Table 1). All patients had manometeric features of failure of relaxation of pelvic floor muscles during attempts to defecate.

**Table 1:** Baseline characteristics and electrophysiological findings of the two patients’ groups

Baseline characteristics and electrophysiological findings	BF group (***n*** = 21 patients) mean ± SD	PTNS group (***n*** = 20 patients) mean ± SD	Test of significance	***P***
Age (years)	40.57 ± 16.97	35.20 ± 13.56	*Z* = − 1.031	0.302
Women^a^	11(52.4)	13(65.0)	*χ*^2^ = 0.672	0.530§
**Anthropometric measurements**				
Weight (kg)	73.09 ± 20.91	67.20 ± 19.88	*Z* = − 0.758	0.449
Height (cm)	162.71 ± 6.45	164.10 ± 7.55	*Z* = − 0.694	0.488
BMI (kg/m^2^)	27.53 ± 7.38	24.78 ± 6.79	*Z* = − 1.422	0.155
**Clinical data**				
Duration of the symptoms (years)	6.47 ± 4.67	5.85 ± 4.97	*Z* = − 0.708	0.479
MOS^b^	5(4–5)	5(4–5)	*Z* = − 1.100	0.271
**Electrophysiological findings**				
**Pudendal nerve status**				
Normal pudendal nerve bilaterally^a^	4(19.0%)	6(30.0%)	*χ*^2^ = 0.767	0.681
Unilateral pudendal neuropathy^a^	4(19.0%)	4(20.0%)		
Bilateral pudendal neuropathy^a^	13(61.9%)	10(50.0%)		
**Needle electromyography**				
EMG evidence of anismus in EAS^a^	16(76.2%)	15(75.0%)	*χ*^2^ = 0.008	0.929
EMG evidence of anismus in PR^a^	21(100%)	20(100%)	NA	NA

*BMI* Body mass index, *MOS* Modified Oxford Scale, *EMG* Electromyography, *EAS* External anal sphincter muscle, *PR* Puborectalis muscle, *BF* Biofeedback pelvic floor muscle training, *PTNS* Posterior tibial nerve electrostimulation, *n* Number of patients, *SD* Standard deviation, *Z* value of Mann-Whitney test, *χ*^2^ value of chi-square test, *NA* not applicable

*P ≤ 0.05 is considered significant

^§^Value of *P* of Fisher’s exact test

^a^Data are represented as number (percentage)

^b^Data are represented as median (range)

Comparison between the baseline assessment and post-intervention assessment of both groups and between both groups in each phase are tabulated in Tables 2 and 3. Regarding post-intervention assessment versus baseline assessment, there were significant differences in all assessed measures in both groups. But, no significant differences were found between the two groups regarding different assessed measures in the pretreatment and post-treatment assessments. There were no patients reported side effects in both therapeutic groups.

**Table 2:** Comparison between the before and after treatment assessments of both groups and between both groups in each phase regarding different assessment questionnaires and time of toileting

Different assessment questionnaires and time of toileting	Pretreatment assessment mean ± SD	Post-treatment assessment mean ± SD	Test of significance^b^	***P***
**MODS**				
BF group (*n* = 21 patients)	12.71 ± 4.73	6.66 ± 6.06	− 4.039	≤ 0.0001*
PTNS group (*n* = 20 patients)	14.05 ± 6.09	10.00 ± 7.73	− 3.749	≤ 0.0001*
**Test of significance**^a^	− 0.693	− 1.596		
***P***	0.488	0.111		
**PAC-QoL**				
BF group (*n* = 21 patients)	44.71 ± 13.98	22.14 ± 16.80	− 4.019	≤ 0.0001*
PTNS group (*n* = 20 patients)	41.05 ± 15.29	31.40 ± 22.76	− 3.467	0.001*
**Test of significance**^a^	− 0.744	− 1.019		
***P***	0.457	0.308		
**Time of toileting (minutes)**				
BF group (*n* = 21 patients)	25.76 ± 16.90	14.47 ± 12.33	− 4.023	≤ 0.0001*
PTNS group (*n* = 20 patients)	29.35 ± 20.65	18.95 ± 16.09	− 3.925	≤ 0.0001*
**Test of significance**^a^	− 0.709	− 0.852		
***P***	0.478	0.394		

*MODS* Modified obstructed defecation score, *BF* biofeedback pelvic floor muscle training, *PTNS* posterior tibial nerve electrostimulation, *n* Number of patients, *PAC-QoL* Patient Assessment of Constipation-Quality of life questionnaire, *SD* standard deviation

*P ≤ 0.05 is considered significant

^a^Value of Mann-Whitney test. It compares the results of the pretreatment assessment between both groups, as well as, the results of the post-treatment assessment between both groups

^b^Value of Wilcoxon signed ranks test. It compares the results of the post-treatment assessment with the results of the pretreatment assessment within each group

**Table 3:** Comparison between the before and after treatment assessments of both groups and between both groups in each phase regarding anal manometry parameters

Anal manometry parameters	Pretreatment assessment mean ± SD	Post-treatment assessment mean ± SD	Test of significance^b^	***P***
**Maximum straining anal pressure (hPa)**				
BF group (*n* = 21 patients)	48.71 ± 15.60	35.42 ± 11.85	− 3.020	0.003*
PTNS group (*n* = 20 patients)	43.70 ± 13.65	36.50 ± 12.33	− 3.416	0.001*
**Test of significance**^a^	− 1.136	− 0.353		
***P***	0.256	0.724		
**Maximum squeezing anal pressure (hPa)**				
BF group (*n* = 21 patients)	65.28 ± 22.82	92.95 ± 28.66	− 4.016	≤ 0.0001*
PTNS group (*n* = 20 patients)	73.45 ± 31.34	85.60 ± 36.86	− 3.683	≤ 0.0001*
**Test of significance**^a^	− 1.201	− 0.366		
***P***	0.230	0.715		

*BF* biofeedback pelvic floor muscle training, *PTNS* posterior tibial nerve electrostimulation, *n* Number of patients, *hPa* hectopascal (it is equal to 100 Pa), *SD* standard deviation

*P ≤ 0.05 is considered significant

^a^Value of Mann-Whitney test. It compares the results of the pretreatment assessment between both groups, as well as, the results of the post-treatment assessment between both groups

^b^Value of Wilcoxon signed ranks test. It compares the results of the post-treatment assessment with the results of the pretreatment assessment within each group

Comparison between the two groups regarding the improvement in different outcome measures are shown in Table 4. Successful outcome was reported in 81% of patients in BF group in comparison to 40% of patients in the PTNS group according to the MODS which was the primary outcome measure. The percentage of patients achieving improvement in all primary and secondary outcome measures except for time of toileting was significantly higher in the BF group in comparison to PTNS group. There were no patients achieved improvement in the maximum straining anal pressure in the PTNS group (Table 4).

**Table 4:** Comparison between both groups regarding improvement in different outcome measures

Outcomes measures	BF group (***n*** = 21 patients) [***n*** (%)]	PTNS group (***n*** = 20 patients) [***n*** (%)]	Test of significance^a^	***P***
**Primary outcome measure**				
MODS improvement	17(81.0)	8(40.0)	7.220	0.011*‡
**Secondary outcome measures**				
PAC-QoL improvement	16(76.2)	6(30.0)	8.789	0.005*
Time of toileting improvement	12(57.1)	8(40.0)	1.205	0.354‡
Maximum straining anal pressure improvement	5(23.8)	0(0)	5.423	0.048*‡

*MODS* Modified obstructed defecation score, *PAC-QoL* Patient Assessment of Constipation-Quality of life questionnaire, *BF* Biofeedback pelvic floor muscle training, *n* Number of patients, *n (%)* Number (percentage) of patients, *PTNS* Posterior tibial nerve electrostimulation, *NA* Not applicable

*P ≤ 0.05 is considered significant

^‡^Value of *P* of Fisher’s exact test

^a^Value of chi-square test

## Discussion

Obstructed defecation constitutes about 1/3 of all patients with constipation. In OD, the patient is unable to defecate in spite of the presence of the natural urge to defecate [31]. Functional OD or anismus responsible for about 25–50% of patients with OD [32, 33]. It results from inappropriate paradoxical contraction or failure of complete relaxation of the EAS and/or PR muscles during attempts defecation [34]. Also, this is known as outlet dysfunction constipation and pelvic floor dyssynergia [13]. Functional OD is considered a maladaptive behavior due to the lack of any associated organic cause for it [35].

Significant differences were found in the assessed measures in both groups between the pretreatment and post-treatment assessments. No significant differences were found between the two groups regarding different assessed measures in the pretreatment and post-treatment assessments. These indicated that both modalities were effective in improving OD severity, improving quality of life with decreasing in the time of toileting. These coincided with several previous studies dealing with functional OD [13–15, 32, 36, 37].

The BF group had a significantly higher percentage of patients achieved improvement in MODS and PAC-QoL in comparison to PTNS. This indicated that BF is superior to PTNS in improving functional OD. Improvement in time of toileting in the BF group was not significantly higher than that of PTNS group. This means that both modalities were effective equally in this issue. This was similar to results of previous studies on patients with functional OD [11, 37, 38].

In the present research, there were significant reduction in the maximum straining anal pressure and improvement in the maximum squeezing anal pressure after treatment in both groups. These indicated that BF and PTNS were effective in improving the anal manometry parameters in OD. The significant decrease in the maximum straining anal pressure is essential as it is the main pathological problem in functional OD [1]. This was similar to previous studies regarding BF [11, 13, 31]. In spite of that, there were no patients achieved improvement (i.e., reduction ≥ 50%) in the maximum straining anal pressure in the PTNS group. This could be because the patients were learned how to relax their EAS and PR muscles during straining to defecate in the BF group only and not PTNS group. However, this was not assessed previously regarding PTNS. The significance increase in the maximum squeezing anal pressure within both groups in the post-treatment assessment was in agreement with previous studies [13, 31, 36]. This is essential to prevent further damage to the pelvic floor muscles secondary to the stretch pudendal neuropathy which is usually associated with OD [27]. The long standing straining during defecation results in excessive stretch of the pudendal nerve with subsequent bilateral pudendal neuropathy [27]. Consequently, the increase in the maximum squeezing anal pressure prevents the late complications of functional OD as pelvic organ prolapse and fecal incontinence [16, 39–41].

The BF group showed successful outcome in 81% of patients in comparison to 40% of patients in the PTNS group according to the primary outcome measure. The study was similar to previous studies regarding the efficacy of BF for treatment of functional OD. Chiarioni et al. reported BF effectiveness in 80% of their patients [42]. Rao et al. achieved improvement in 79% of their patients [43]. Lembo et al. reported improvement in 82% of their patients [44]. Wiesel et al. reported improvement in 79% of their participants [45]. Kuang et al. reported BF efficacy to be 76% [38].

The mechanism of action of BF is to coordinate the activity of EAS and other pelvic floor muscles with abdominal muscles for complete defecation [31, 46]. BF is a form of cognitive behavioral therapy. The contraction and relaxation of the anal sphincter muscles are converted into visual and auditory signals through which the patients could learn how to control the pathological function [47]. The patients learn how to relax their EAS and PR muscles voluntarily during straining and attempts to defecate. This is done with the aid of BF pelvic floor muscle training [48]. BF training allows information of physiological processes to be converted into visual and auditory signals which allow the patients to learn and acquire the ability to control their disturbed defection process [49]. This requires patient motivation, orientation and concentration with active participation in the treatment of themselves [50]. The treating physician who do the BF session is only an assistant to the patient in the therapy. The improvement needs patient active participation during the BF session [10, 38].

The current study was like previous studies regarding the efficacy of PTNS in treatment of functional OD [15, 36, 37]. PTNS is a form of peripheral neuromodulation. It acts by modulation of the ascending neuronal pathways to the sensory cortex [51, 52]. Bilateral PTNS was found to be more effective than unilateral PTNS. This could be due to the activation of a greater number of neuronal afferent pathways [15]. During the neuromodulation session, the patient is completely passive. No need for any active participation of the patient during the session [53, 54]. The effect of neuromodulation takes place in a subconscious level that the patient could not recognize it except by the observation that the OD is gradually improved [15, 51, 52]. The neuromodulation does not need any reinforcement during the therapy session as in BF session. The PTNS was the preferred method in some patients who preferred not to expose themselves during the BF session. However, other patients preferred BF sessions because they were not convenient with PTNS which was applied in the leg region far away from the pelvic floor.

No side effects occurred in any patient in both therapeutic groups. This was similar to the literature in which BF and PTNS were considered safe physical modalities and not associated with any side effects [13, 15, 16, 30, 31, 36–38, 54].

In the study, the overall improvement was towards the BF group. The significantly superior effect of BF in comparison to PTNS in treatment of functional OD could make PTNS to be considered as a second line therapy after failure of BF therapy [8].

The study results were like other researches that assessed the efficacy of BF versus PTNS in FI and in overactive bladder in spite of different pelvic floor medical problems [29, 30, 55]. The higher success rate of BF group could be due to the active correction of the functional disturbance in the EAS and PR muscles associated with the high motivation in the participated patients [13, 56, 57]. Good cooperation between the patients and the performing physician is critical for the success of BF [58, 59]. This could not be seen in the PTNS in which the patients did not actively learn how to relax their EAS and PR muscles during attempt defecation.

The combination between two different modalities which act through two different mechanisms of action is considered a good choice for treatment as applied for other pelvic floor sphincteric disorders [29, 30, 55]. It is suspected to be the most effective method in combination with health education, dietary modification and life style modification in the treatment of functional OD. The combination therapy is usually more effective than monotherapy [29, 30, 38, 55]. This is suspected to decrease the duration of treatment, increase the patient satisfaction to the therapy, decrease the failure rate, and decrease the rate of more invasive therapeutic modalities as surgery for patients with intractable functional OD [2, 10].

## Limitations

First limitation, blinding protocol was not applied in the current study because of the differences in the treatment procedure and modalities between the two treatment groups. This could be a source of bias in the current study. Second limitation, the limited number of participants. This could be due to the large scope of exclusion criteria. Further researches on a larger number of patients is recommended. Third limitation, the short-term follow-up of the patients. The study aimed to assess the short-term efficacy of BF versus PTNS. The long-term effects of both of them were assessed in many previous studies and this was out of the scope of the current study [31, 32]. Fourth limitation, the study included patient with only functional OD and did not include patients with mechanical OD. Further researches assessing the efficacy of BF versus PTNS in the treatment of patients with functional OD associated with mechanical OD is recommended. Fifth limitation, the study did not include a group of patient who received a combined therapy of BF with PTNS. Further researches assessing this issue is recommended to clarify the significance of combined therapy in comparison to monotherapy. Sixth limitation, the investigation had been done in one medical center, consequently the generalizability of the obtained results must be taken with precautions.

## Conclusion

In conclusion, both BF and PTNS are considered effective methods in the treatment of functional OD. However, BF seems to be more effective and superior in comparison to PTNS. PTNS could be combined with BF or considered as a second line therapy after failure of BF.

## Data Availability

The datasets used and/or analyzed during the current study are available from the corresponding author on reasonable request.

## Abbreviations

BF: Biofeedback pelvic floor muscle training
BMI: Body mass index
EAS: External anal sphincter
EMG: Electromyography
hPa: Hectopascal
MODS: Modified obstructed defecation score
MOS: Modified Oxford Scale
N: Number of patients
NA: Not applicable
OD: Obstructed defecation
PAC-QoL: Patient Assessment of Constipation-Quality of Life questionnaire
PR: Puborectalis
PTNS: Posterior tibial nerve electrostimulation
SD: Standard deviation
*χ*^2^: Chi-square test
Z: Mann-Whitney test
